# Association of aerobic capacity and handgrip strength in obese and non-obese children aged 10–15 years in Riyadh region, KSA–a cross sectional study

**DOI:** 10.1038/s41598-026-43515-7

**Published:** 2026-03-17

**Authors:** Gopal Nambi, Mshari Alghadier, Arul Vellaiyan, Shahul Hameed Pakkir Mohamed, Vijayamurugan Eswaramoorthi, Ramprasad Muthukrishnan

**Affiliations:** 1 Department of Health and Rehabilitation Sciences, College of Applied Medical Sciences, Prince Sattam bin Abdulaziz University, Al-Kharj, Saudi Arabia; 2 Department of Maternal and Child Health Nursing, College of Nursing, Prince Sattam bin Abdulaziz University, Al-Kharj, Saudi Arabia; 3 Department of Health Rehabilitation Sciences, Faculty of Applied Medical Sciences, University of Tabuk, Tabuk, Saudi Arabia; 4 Saveetha College of Physiotherapy, Saveetha Institute of Medical and Technical Sciences (Deemed to the University), Chennai, 600077 TamilNadu India; 5 Department of Physiotherapy, Faculty of Pharmacy and Health Sciences, Universiti Kuala Lumpur, Royal College of Medicine Perak, Ipoh, 30450 Malaysia; 6 Department of Physiotherapy, College of Health Sciences, Gulf Medical University, Ajman, UAE

**Keywords:** Aerobic capacity, Handgrip strength, Obesity, Children, Cross-sectional study, Health care, Medical research, Physiology, Risk factors

## Abstract

Childhood obesity is associated with impaired physical fitness and increased future cardiometabolic risk. Aerobic capacity and muscular strength are key components of health-related fitness; however, their interrelationship across weight status remains underexplored in Middle Eastern pediatric populations. This study aimed to examine the association between aerobic capacity and muscular strength among obese and non-obese children aged 10–15 years in the Riyadh region, Kingdom of Saudi Arabia. A cross-sectional study was conducted among 200 children (52% boys and 48% girls) selected from screened government and private school students in Riyadh, comprising 100 obese and 100 non-obese participants for balanced group comparison. Obesity was defined using World Health Organization age- and sex-specific BMI percentiles. Aerobic capacity was assessed using the 20-m shuttle run test, with estimated VO2max calculated from performance. Muscular strength was measured using handgrip dynamometry, and relative strength was derived by normalizing grip strength to body weight. Group comparisons were performed using independent t-tests. Associations between aerobic capacity and muscular strength were analyzed using correlation and multivariable linear regression adjusting for age, sex, and BMI. Obese children demonstrated significantly lower shuttle run performance and estimated VO2max compared with non-obese children (p < 0.001). Absolute handgrip strength was higher in obese children (p = 0.01), whereas relative muscular strength was significantly lower (p < 0.001). Aerobic capacity showed moderate to strong positive correlations with both absolute and relative muscular strength in both groups, with stronger associations observed for relative strength. In multivariable regression analyses conducted using separate models to avoid mathematical coupling, relative muscular strength independently predicted VO2max after adjusting for age and sex (β = 0.47, p < 0.001), explaining 52% of variance. In a separate adiposity model, BMI was inversely associated with aerobic capacity (β = −0.51, p < 0.001), explaining 56% of variance. Obese children aged 10–15 years exhibited reduced aerobic capacity and lower relative muscular strength despite higher absolute strength. Both adiposity and functional muscular strength were independently associated with aerobic fitness when modeled separately. The stronger predictive value of relative muscular strength highlights the importance of improving strength relative to body mass, alongside aerobic conditioning, in pediatric obesity prevention and intervention programs.

## Introduction

Childhood obesity represents one of the most pressing public health issues of the 21 st century, with its prevalence rising significantly in both high-income and low- to middle-income countries over the past few decades^1^. The global escalation in pediatric obesity has been linked to adverse health outcomes, including early onset of metabolic syndrome, cardiovascular abnormalities, and reduced physical fitness, all of which can persist into adulthood. In children aged 10–15 years, obesity is frequently associated with diminished components of physical fitness, such as aerobic capacity and muscular strength, underscoring the importance of investigating these relationships to inform preventive health strategies^2^.

Obesity is inversely associated with aerobic capacity, and muscular strength is an important component of physical function. In interpreting strength–fitness relationships, it is also important to consider the biomechanical context of performance tests. Weight-bearing tasks such as shuttle runs involve integrated neuromuscular coordination and whole-body mechanical work against gravity, which can markedly influence metabolic cost and aerobic responses compared with non-weight-bearing measures focused on isolated muscle groups (e.g., handgrip) that primarily reflect local force capacity rather than systemic locomotive performance^3^. Recent work suggests that weight-bearing and non-weight-bearing strength assessments differ in their mechanical demands and physiological correlates, which may partly account for variation in associations with aerobic capacity across studies^4^.

Aerobic capacity, commonly quantified through maximal oxygen uptake (VO₂max) or field-based measures such as shuttle run performance, is a core indicator of cardiorespiratory fitness and reflects the integrated function of the cardiovascular, pulmonary, and muscular systems during sustained exercise. It reflects the efficiency of the cardiovascular, pulmonary, and muscular systems in transporting and utilizing oxygen during sustained physical activity^5^. In children and adolescents, higher aerobic capacity correlates with favorable cardio metabolic profiles, including improved lipid levels, blood pressure regulation, and insulin sensitivity^6^. Conversely, lower aerobic fitness has been consistently reported in children with obesity compared with their normal-weight peers, a disparity that may be attributed to increased body mass, altered biomechanics, and reduced habitual physical activity levels^7^.

Muscular strength represents another essential dimension of health-related physical fitness, contributing to daily functional tasks, motor skill development, metabolic health, and overall physical performance^8^. Strength development during childhood not only facilitates participation in physical activities but also supports healthy musculoskeletal growth. Although children with obesity may demonstrate greater absolute muscle mass due to increased body weight, evidence indicates that their relative muscular strength — strength adjusted for body mass — tends to be lower than that of non-obese children^9^. This relative deficit can compromise movement efficiency and endurance, potentially creating barriers to physical activity and further exacerbating sedentary behaviors.

The association between aerobic capacity and muscular strength in children is complex and influenced by physiological, behavioral, and developmental factors. Several studies have suggested a positive correlation between these two components of fitness, implying that greater muscular strength may support enhanced aerobic performance through improved movement efficiency and neuromuscular coordination^10^. Nonetheless, the presence of excessive adiposity may attenuate this relationship. Obesity introduces greater mechanical load and metabolic demand during both strength and aerobic activities, which may alter typical associations observed in normal-weight populations^11^. Understanding how obesity modifies the interplay between aerobic capacity and muscular strength is therefore essential for developing targeted interventions.

Cross-sectional research has provided valuable insights into fitness differences between obese and non-obese children; however, findings have not always been consistent, largely due to variations in sample age ranges, fitness assessment methods, and demographic contexts^9,12^. Moreover, the majority of existing research originates from Western populations, limiting generalizability to other regions that may differ in lifestyle patterns, cultural attitudes toward physical activity, and obesity prevalence^2,13^. There is a notable gap in the literature regarding pediatric fitness profiles in Middle Eastern countries, particularly within the Kingdom of Saudi Arabia (KSA).

Saudi Arabia has experienced rapid socioeconomic transformation over recent decades, accompanied by shifts in diet, physical activity, and daily living patterns. Such shifts have contributed to increased rates of overweight and obesity among children and adolescents, especially in urban settings such as the Riyadh region^14^. Studies conducted within the Saudi pediatric population report high prevalence estimates of overweight and obesity, raising concerns about associated declines in physical fitness and increased future health burdens^15^. Despite this recognized trend, research focusing on specific aspects of physical fitness — and the relationships among them — remains limited.

Recent Saudi-based investigations have begun to address components of pediatric physical fitness. Al-Asiri Z. A et al. examined the relationship between BMI and health-related physical fitness in Saudi girls aged 8–15 years, reporting inverse associations between adiposity and selected fitness components^16^. More recently, Alonazi A. et al. evaluated gender differences in lower-limb strength and endurance among Saudi adolescents and reported that BMI played a limited role in explaining strength performance^17^. Additionally, Alotaibi S. et al. demonstrated that handgrip strength predicts leg power but not cardiorespiratory fitness in Saudi children^18^. However, these studies primarily focused on isolated fitness components or gender comparisons and did not specifically examine the interrelationship between aerobic capacity and handgrip strength across obesity categories using adjusted multivariable modeling. Furthermore, none of the available Saudi studies have evaluated whether relative muscular strength independently predicts aerobic capacity when adiposity-related collinearity is statistically controlled. Therefore, a gap remains in understanding how muscular strength and aerobic capacity interact in obese versus non-obese Saudi children.Investigation of aerobic capacity and muscular strength in Saudi children gains particular importance in the context of the 10–15-year age range, a period marked by rapid growth, pubertal maturation, and the establishment of long-term health behaviors^19^. This developmental phase presents a critical window in which physical fitness levels may influence lifelong health trajectories. Examination of how obesity interacts with two key fitness dimensions during this period can therefore yield insights that inform effective school- and community-based health promotion programs.

Despite the rising prevalence of childhood obesity in the Kingdom of Saudi Arabia, limited data exist regarding the interrelationship between aerobic capacity and handgrip strength among Saudi school-aged children. Most available evidence originates from Western populations, potentially limiting generalizability due to differences in lifestyle, sociocultural norms, and physical activity patterns. By examining these associations in children aged 10–15 years in the Riyadh region, this study provides region-specific evidence from a Middle Eastern population that remains underrepresented in pediatric fitness research. Such data are essential for informing culturally appropriate school-based and community-level obesity prevention strategies in Saudi Arabia.

Given the emerging Saudi literature on pediatric fitness, there remains a need to clarify whether muscular strength—particularly strength relative to body mass—is independently associated with aerobic capacity across obesity categories. Unlike prior Saudi studies that examined gender differences, isolated BMI associations, or predictive relationships between handgrip and leg power, the present study investigates the strength–aerobic capacity relationship stratified by obesity status and employs separate regression models to avoid mathematical coupling between BMI and relative strength. This analytical distinction provides a more precise understanding of the independent contributions of adiposity and functional muscular performance to aerobic fitness.

## Methods

### Study design and setting

This study employed a cross-sectional observational design, conducted in accordance with the Strengthening the Reporting of Observational Studies in Epidemiology (STROBE) guidelines. The study was carried out in selected government and private schools in the Riyadh region, Kingdom of Saudi Arabia (KSA). Data collection was performed over a defined study period (Mar 2025 to Oct 2025), during regular school hours in designated physical education or assessment areas within the school premises.

### Study population

The study population comprised children aged 10–15 years enrolled in participating schools in the Riyadh region. This age group was selected as it represents a critical developmental period characterized by rapid physical growth, pubertal changes, and evolving physical fitness patterns.

### Sampling technique

A multistage sampling strategy was employed. In the first stage, 12 government and private schools across different districts of the Riyadh region were invited to participate to enhance geographic representation. Nine schools (5 government and 4 private) agreed to participate. In the second stage, all students aged 10–15 years within selected schools were invited to undergo anthropometric screening following parental consent. Height and weight were measured, and BMI percentiles were calculated using WHO age- and sex-specific growth reference standards. Students were then categorized into BMI groups: obese (≥ 95th percentile) and non-obese (< 85th percentile). Children classified as overweight (85th–94th percentile) were excluded to ensure clear group distinction. From the eligible screened pool, participants were selected using stratified random sampling within BMI categories, stratified further by age and sex to maintain proportional representation. A balanced analytical sample of 100 obese and 100 non-obese participants was selected to ensure adequate statistical power for between-group comparisons and multivariable analyses. Equal group allocation was implemented for analytical comparability and does not reflect the natural BMI distribution within the source population.

### Eligibility criteria

Children aged 10–15 years enrolled in selected schools in the Riyadh region were eligible to participate in this study. Participants were required to be apparently healthy and physically capable of performing the required fitness assessments. Written informed consent was obtained from parents or legal guardians, along with assent from the children prior to participation. Children were excluded if they had any known cardiovascular, respiratory, neurological, musculoskeletal, endocrine, or metabolic disorders that could affect physical performance; if they were taking medications known to influence exercise capacity; or if they had an acute illness or injury at the time of data collection that could limit safe participation in the assessments.

### Sample size

The sample size was calculated using G*Power software (version 3.1.9.7, Heinrich Heine University, Düsseldorf, Germany) based on detecting a moderate effect size (Cohen’s d = 0.50) for differences in aerobic capacity (VO_₂max_) between obese and non-obese children, as reported in previous pediatric fitness studies. Assuming a two-tailed independent samples t-test, an alpha level of 0.05, and a statistical power of 80%, the minimum required sample size was estimated to be 64 participants per group. To enhance statistical robustness, allow for subgroup analyses, and account for potential incomplete data, the sample size was increased to 100 participants per group. Therefore, a total of 200 children (100 obese and 100 non-obese) were included in the final analysis.

### Anthropometric measurements

Anthropometric assessments were conducted following standardized protocols. Body weight was measured to the nearest 0.1 kg using a calibrated digital weighing scale, with participants wearing light clothing and no shoes. Height was measured to the nearest 0.1 cm using a portable stadiometer, with participants standing upright in the Frankfurt plane. Body mass index (BMI) was calculated as weight (kg) divided by height squared (m²). Participants were classified as obese or non-obese using age- and sex-specific BMI percentiles based on World Health Organization (WHO) growth reference standards, with obesity defined as BMI ≥ 95th percentile.

### Assessment of aerobic capacity

Aerobic capacity was assessed using a validated field-based test suitable for children aged 10–15 years, such as the 20-meter shuttle run test (PACER). Participants were instructed to run back and forth between two lines spaced 20 meters apart, following audio signals that progressively increased in speed. The test was terminated when the participant failed to reach the line on two consecutive occasions or voluntarily stopped due to fatigue. Performance was recorded as the total number of completed laps, and estimated VO_2max_was calculated using the Léger prediction equation^20^:

$$VO_{{2\max }} \left( {ml \cdot kg^{{ - 1}} \cdot min^{{ - 1}} } \right) = 31.025 + 3.238 \times speed - 3.248 \times age + 0.1536 \times speed \times age$$

Where speed represents the final running speed (km·h⁻¹) achieved during the test. This equation has been widely validated in pediatric populations and is commonly used in field-based fitness assessments. Standardized verbal encouragement was provided, and safety precautions were observed throughout testing. The Léger equation has been widely validated in general pediatric populations; however, it was not specifically derived from or definitively validated in children with BMI ≥ 95th percentile. As with many field-based prediction models, accuracy may decline at extremes of body mass due to altered biomechanics and movement economy. Therefore, estimated VO₂max values in obese participants should be interpreted with appropriate caution.

### Assessment of muscular strength

Muscular strength was evaluated using handgrip strength, a reliable and widely used indicator of overall muscular strength in children. Handgrip strength was measured using a calibrated handheld dynamometer. Participants performed the test in a standing position with the arm by the side and elbow fully extended. Two trials were conducted for each hand, with a rest interval between trials. The highest value obtained for each hand was recorded, and absolute handgrip strength (kg) was calculated as the mean of the highest right- and left-hand values to represent bilateral maximal strength. Relative muscular strength was calculated by normalizing absolute handgrip strength to body weight (kg/kg) to allow meaningful comparison between obese and non-obese participants.

### Data collection procedure

All assessments were conducted during regular school days between 8:30 AM and 11:30 AM to minimize variability related to circadian rhythm and daily activity fluctuations. Each participating school was assessed within a consistent morning time window to ensure temporal standardization across participants. Participants were instructed to avoid vigorous physical activity on the morning of testing and to consume their usual breakfast to reduce acute metabolic variability.

The testing protocol followed a standardized sequence for all participants:

1. Anthropometric measurements (height and weight).
2. Handgrip strength assessment.
3. 20-meter shuttle run test.

This fixed order was selected to prevent fatigue from aerobic testing from influencing handgrip performance. A rest interval of at least 5 min was provided between handgrip trials and prior to the shuttle run test. Standardized verbal instructions and encouragement were given during all assessments. Although the testing sequence was not randomized, it was uniformly applied to all participants to ensure consistency and reduce procedural variability.

### Statistical analysis

Data were analyzed using a statistical software package (e.g., SPSS version 31.0.1.0). Descriptive statistics were calculated for all variables and presented as mean ± standard deviation or frequencies and percentages, as appropriate. Normality of data distribution was assessed using the Shapiro–Wilk test. Comparisons between obese and non-obese groups were performed using independent t-tests or Mann–Whitney U tests for continuous variables and chi-square tests for categorical variables. The association between aerobic capacity and muscular strength was examined using Pearson or Spearman correlation coefficients, depending on data distribution. For visual interpretation of associations, scatter plots with fitted linear regression lines and 95% confidence intervals were generated stratified by obesity status.

Multivariable linear regression analyses were conducted in the pooled sample (*n* = 200) to examine independent predictors of VO_₂max_ while adjusting for age and sex. Because relative muscular strength is derived from body weight and BMI is also weight-dependent, these variables were not entered simultaneously to avoid mathematical coupling and multicollinearity. Separate models were therefore constructed: one including relative muscular strength, and another including BMI. Standardized beta coefficients (β), 95% confidence intervals (CI), and p-values were reported. Model fit was evaluated using R² and adjusted R², and multicollinearity was assessed using variance inflation factor (VIF). Regression assumptions were examined using standard diagnostic procedures. A sensitivity analysis was performed replacing relative muscular strength with absolute handgrip strength while adjusting for BMI, age, and sex. Statistical significance was set at *p* < 0.05.

### Ethical considerations

Ethical approval was obtained from the appropriate Institutional Review Board (IRB) of the affiliated institution. Permission was also secured from school authorities prior to data collection. Participation was voluntary, and confidentiality of participant data was strictly maintained. All procedures were conducted in accordance with the principles of the Declaration of Helsinki.

## Results

### Participant characteristics

A total of 200 children (52% boys and 48% girls) participated in the study. Of these, 100 children were classified as obese and 100 as non-obese based on age- and sex-specific BMI percentiles. The mean age of the participants was 12.6 ± 1.7 years, with no significant difference between obese and non-obese groups (*p* = 0.42). Sex distribution was comparable between groups (*p* = 0.68). There were no statistically significant differences in height between obese and non-obese children (153.1 ± 9.5 cm vs. 151.7 ± 10.1 cm; *p* = 0.31). As expected, obese children had significantly greater body weight **(**63.8 ± 11.6 kg**)** and BMI **(**27.1 ± 3.2 kg/m²) compared with non-obese children **(**41.4 ± 8.9 kg and 18.9 ± 2.4 kg/m², respectively; *p* < 0.001 for both). BMI percentile was significantly higher in the obese group (median 96.5; IQR 95.3–98.1) compared with the non-obese group (median 52.0; IQR 39.5–68.4; *p* < 0.001) (Table 1).

**Table 1:** Demographic and anthropometric characteristics of the study participants.

Variable	Total (n = 200)	Obese (n = 100)	Non-Obese (n = 100)	p-value
Age (years), mean ± SD	12.6 ± 1.7	12.7 ± 1.6	12.5 ± 1.8	0.42
Sex, n (%)				0.68
Male	104 (52%)	52 (52%)	52 (52%)	
Female	96 (48%)	48 (48%)	48 (48%)	
Height (cm), mean ± SD	152.4 ± 9.8	153.1 ± 9.5	151.7 ± 10.1	0.31
Weight (kg), mean ± SD	52.6 ± 14.3	63.8 ± 11.6	41.4 ± 8.9	<0.001
BMI (kg/m²), mean ± SD	22.5 ± 4.8	27.1 ± 3.2	18.9 ± 2.4	<0.001
BMI Percentile, (median IQR)	78.5 (56.2–94.1)	96.5 (95.3–98.1)	52.0 (39.5–68.4)	<0.001

### Comparison of aerobic capacity

Aerobic capacity outcomes differed significantly between obese and non-obese participants. Performance in the 20-m shuttle run test was markedly lower in obese children, who completed a mean of 25.4 ± 8.6 laps, compared with 39.8 ± 10.2 laps in non-obese children (*p* < 0.001). Similarly, estimated VO_₂max_ was significantly lower in the obese group **(**38.2 ± 4.5 ml·kg⁻¹·min⁻¹) compared with the non-obese group **(**46.9 ± 5.1 ml·kg⁻¹·min⁻¹**)**, with a mean difference of 8.7 ml·kg⁻¹·min⁻¹ (*p* < 0.001) (Table 2).

**Table 2:** Comparison of aerobic capacity and muscular strength between obese and non-obese children.

Variable	Obese (n = 100)	Non-Obese (n = 100)	Mean Difference (95% CI)	Cohen’s d	p-value
20 m Shuttle Run (laps)	25.4 ± 8.6	39.8 ± 10.2	−14.4 (−17.0 to −11.8)	1.55	<0.001*
Estimated VO₂max (ml·kg⁻¹·min⁻¹)	38.2 ± 4.5	46.9 ± 5.1	−8.7 (−10.0 to −7.4)	1.80	<0.001*
Handgrip Strength – Right (kg)	23.8 ± 6.1	21.4 ± 5.8	+2.4 (0.6 to 4.2)	0.40	0.01*
Handgrip Strength – Left (kg)	22.9 ± 5.9	20.7 ± 5.6	+2.2 (0.4 to 4.0)	0.38	0.02*
Absolute Handgrip Strength (kg)	23.4 ± 5.7	21.1 ± 5.5	+2.3 (0.5 to 4.1)	0.41	0.01*
Relative Handgrip Strength (kg/kg)	0.37 ± 0.08	0.51 ± 0.09	−0.14 (−0.16 to −0.12)	1.63	<0.001*

Values are presented as mean ± standard deviation (SD), number (%), n - number, ml – milliliter, kg – Kilogram, min – minute. Cohen’s d represents standardized effect size (small = 0.2, medium = 0.5, large ≥ 0.8).

### Comparison of muscular strength

In contrast to aerobic capacity, absolute muscular strength was significantly higher in obese children. Mean right-hand grip strength was 23.8 ± 6.1 kg in obese participants versus 21.4 ± 5.8 kg in non-obese participants (*p* = 0.01). Similar findings were observed for left-hand grip strength (22.9 ± 5.9 kg vs. 20.7 ± 5.6 kg; *p* = 0.02). Overall absolute handgrip strength was higher in obese children (23.4 ± 5.7 kg) compared with non-obese children (21.1 ± 5.5 kg; *p* = 0.01) (Table 2). However, when muscular strength was normalized to body weight, relative muscular strength was significantly lower in obese children (0.37 ± 0.08 kg/kg) than in non-obese children (0.51 ± 0.09 kg/kg; *p* < 0.001).

### Association between aerobic capacity and muscular strength

**Fig. 1:**
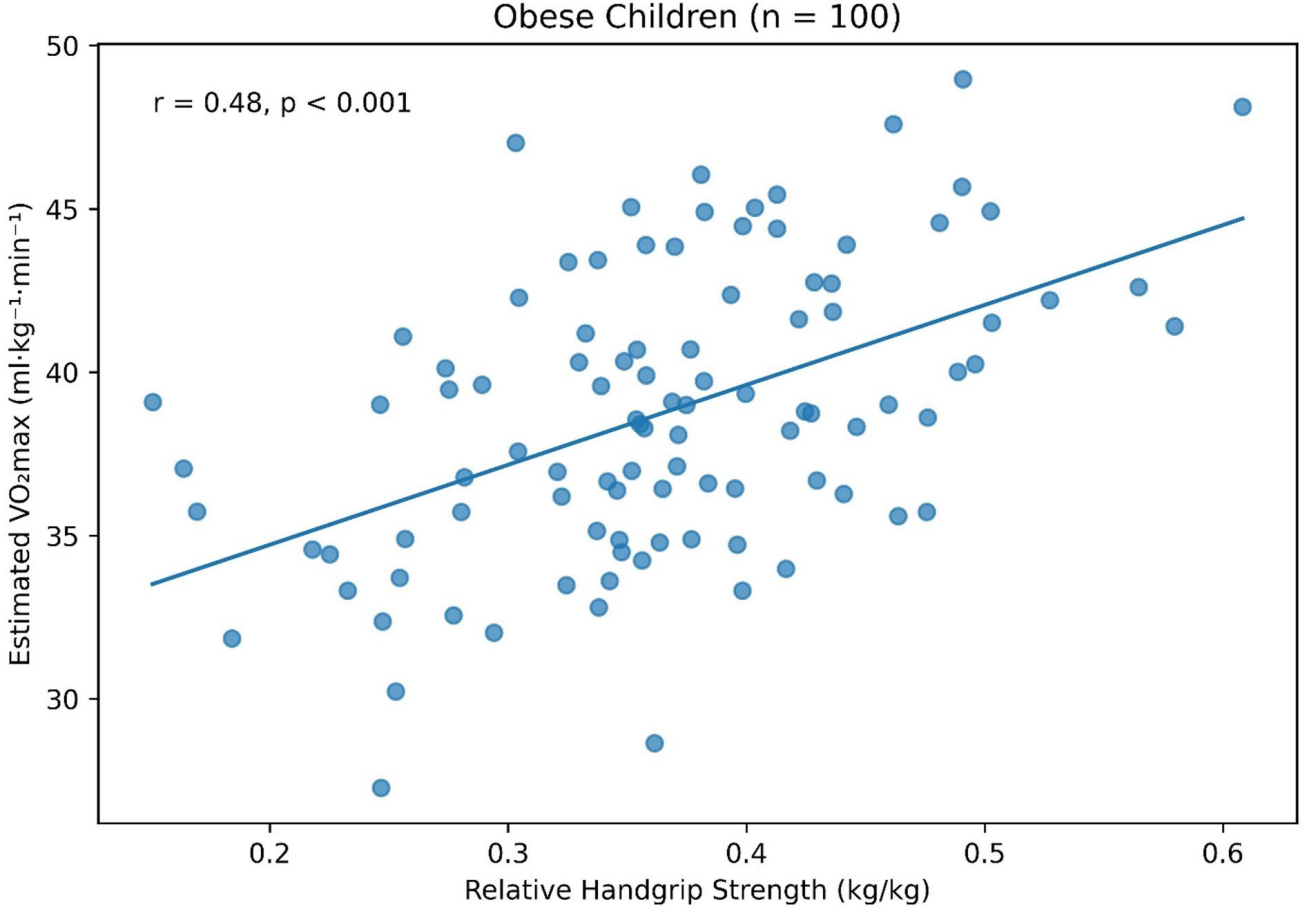
Scatter plots illustrating the association between estimated VO₂max and relative handgrip strength in obese and non-obese children.

Correlation analysis demonstrated a positive association between aerobic capacity and muscular strength in both groups. In obese children, estimated VO_₂max_ was moderately correlated with absolute handgrip strength (*r* = 0.32, *p* = 0.002) and more strongly correlated with relative handgrip strength (*r* = 0.48, *p* < 0.001). Similar patterns were observed for shuttle run performance, which showed significant correlations with both absolute (*r* = 0.29, *p* = 0.004) and relative muscular strength (*r* = 0.46, *p* < 0.001). Among non-obese children, stronger associations were observed. VO_₂max_ demonstrated significant correlations with absolute (*r* = 0.41, *p* < 0.001) and relative handgrip strength (*r* = 0.56, *p* < 0.001). Shuttle run laps were also positively correlated with absolute (*r* = 0.38, *p* < 0.001) and relative muscular strength (*r* = 0.54, *p* < 0.001) (Table 3). The relationship between estimated VO_₂max_ and relative handgrip strength stratified by obesity status is illustrated in Fig. 1, demonstrating a stronger positive association in non-obese children compared with obese children.

**Table 3:** Association between aerobic capacity and muscular strength in obese and non-obese children.

Association	Obese (r, p-value)	Non-Obese (r, p-value)
VO₂max vs Absolute Handgrip Strength	0.32, p = 0.002	0.41, p < 0.001
VO₂max vs Relative Handgrip Strength	0.48, p < 0.001	0.56, p < 0.001
Shuttle Run Laps vs Absolute Handgrip Strength	0.29, p = 0.004	0.38, p < 0.001
Shuttle Run Laps vs Relative Handgrip Strength	0.46, p < 0.001	0.54, p < 0.001

Values represent Pearson correlation coefficients (r) with corresponding p-values. Correlation strength was interpreted as weak (<0.30), moderate (0.30–0.49), and strong (≥0.50).

### Multivariable analysis

Multivariable linear regression analyses were performed on the pooled sample (*n* = 200). In Model 1 (relative muscular strength model), after adjusting for age and sex, relative muscular strength emerged as a significant independent predictor of VO_₂max_ (β = 0.47, *p* < 0.001). Male sex (β = 0.20, *p* = 0.003) and increasing age (β = 0.17, *p* = 0.012) were also positively associated with aerobic capacity. The model demonstrated good explanatory power (R² = 0.52; Adjusted R² = 0.50). Multicollinearity diagnostics indicated acceptable levels (VIF range: 1.12–1.34; tolerance range: 0.74–0.89), indicating no problematic collinearity.

In Model 2 (adiposity model), BMI was entered instead of relative muscular strength. BMI demonstrated a strong inverse association with VO_₂max_ (β = −0.51, *p* < 0.001) after adjusting for age and sex. Male sex (β = 0.19, *p* = 0.004) and age (β = 0.16, *p* = 0.015) remained significant predictors. This model explained 56% of the variance in aerobic capacity (R² = 0.56; Adjusted R² = 0.54). Collinearity statistics remained within acceptable limits (VIF range: 1.18–1.42; tolerance range: 0.70–0.85).

A sensitivity analysis replacing relative muscular strength with absolute handgrip strength while adjusting for BMI revealed that absolute strength remained a modest but significant predictor of VO_₂max_ (β = 0.21, *p* = 0.009), though the association was weaker compared with the relative strength model.

These findings indicate that both adiposity and muscular fitness are independently associated with aerobic capacity; however, statistical modeling confirms that BMI and relative muscular strength should not be included simultaneously due to shared variance related to body mass, Table 4.

**Table 4:** Multivariable linear regression models predicting VO₂max (ml·kg⁻¹·min⁻¹) with model fit and collinearity diagnostics in the pooled sample (n = 200).

Predictor Variable	Standardized β	95% CI	p-value
Model 1: Relative Muscular Strength Model			
Age (years)	0.17	0.08 to 0.30	0.012
Sex (Male)	0.20	0.14 to 0.36	0.003
Relative Muscular Strength (kg/kg)	0.47	0.31 to 0.63	<0.001
R² = 0.52; Adjusted R² = 0.50			
VIF range: 1.12–1.34			
Model 2: Adiposity Model			
Age (years)	0.16	0.07 to 0.29	0.015
Sex (Male)	0.19	0.13 to 0.35	0.004
BMI (kg/m²)	−0.51	−0.67 to −0.35	<0.001
R² = 0.56; Adjusted R² = 0.54			
VIF range: 1.18–1.42			

Multivariable linear regression models predicting VO₂max (ml·kg⁻¹·min⁻¹) in the pooled sample (n = 200). Standardized beta coefficients (β) with 95% confidence intervals (CI) are presented. R² and adjusted R² indicate model explanatory power. Variance inflation factor (VIF) values are provided to assess multicollinearity (VIF < 5 considered acceptable). Kg – Kilogram, m – meter, BMI – Body mass index

## Discussion

The present cross-sectional study investigated the association between aerobic capacity and muscular strength among obese and non-obese children in the Riyadh region, Kingdom of Saudi Arabia. The main findings indicate that obese children exhibited significantly lower aerobic capacity, as evidenced by reduced shuttle run performance and estimated VO₂max, compared with their non-obese counterparts. Conversely, obese children demonstrated higher absolute muscular strength but significantly lower relative muscular strength when normalized for body weight. Furthermore, aerobic capacity was positively associated with muscular strength in both groups, with stronger associations observed for relative muscular strength.

### Aerobic capacity in obese and non-obese children

Our findings of reduced aerobic capacity among obese children are consistent with a substantial body of literature demonstrating an inverse relationship between adiposity and aerobic capacity in children and adolescents^1,2^. Previous studies using the 20-m shuttle run test have consistently reported lower endurance performance and estimated VO₂max in obese children due to increased mechanical load, reduced movement efficiency, and higher energy expenditure during weight-bearing activities^5,6^. An important biomechanical consideration is that the 20-m shuttle run represents a repeated change-of-direction (COD) task requiring cyclical braking and propulsive phases. During each 180° turn, participants must generate substantial eccentric force to decelerate forward momentum (braking phase), followed by rapid concentric force production to re-accelerate in the opposite direction. These transitions are characterized by elevated ground reaction forces, prolonged ground contact times, and increased neuromuscular demand. In children with obesity, greater body mass amplifies inertial load, thereby increasing the mechanical work required during both deceleration and re-acceleration. This elevation in mechanical demand directly inflates the metabolic cost of the task, as greater oxygen consumption is required to repeatedly control and propel excess mass. Consequently, shuttle run performance in this population reflects not only cardiorespiratory fitness but also load-bearing tolerance and neuromuscular braking efficiency. Reduced performance in obese children may therefore represent a composite limitation involving aerobic capacity, impaired COD mechanics, and increased energy expenditure per transition, rather than purely central cardiovascular restriction^3^.

In addition to the biomechanical constraints inherent in repeated change-of-direction tasks, the estimation of VO₂max using the Léger prediction equation introduces further methodological considerations in obese youth. The equation is based on final shuttle run speed, a performance-derived variable that is intrinsically influenced by body mass^20^. Consequently, children with high adiposity may obtain lower estimated VO₂max values not solely due to reduced physiological aerobic capacity, but also due to the increased metabolic and mechanical cost of accelerating and decelerating excess mass. Therefore, in obese participants, reduced estimated VO₂max may represent a composite of true cardiorespiratory limitation and body-mass-related performance constraints. This distinction is important when interpreting aerobic fitness differences between BMI groups.

Low aerobic capacity during childhood is clinically significant, as cardiorespiratory fitness is a strong and independent predictor of future cardiovascular and metabolic health^6^. Longitudinal evidence suggests that children with low aerobic fitness are more likely to develop insulin resistance, dyslipidemia, and hypertension later in life, regardless of weight status^8^. Given the rising prevalence of childhood obesity in Saudi Arabia, particularly in urban regions such as Riyadh, the observed reduction in aerobic fitness among obese children highlights an urgent need for early preventive strategies^9^.

### Methodological considerations regarding strength normalization

Although normalizing muscular strength by body mass (kg/kg) is a commonly applied method in pediatric field-based research, the relationship between body mass and strength expression is complex and often nonlinear. Simple ratio scaling may inadequately adjust for differences in body size, particularly when height and other body dimensions contribute meaningfully to strength performance. Recent methodological studies in youth strength normalization using allometric scaling show that optimal normalization may require consideration of multiple body size dimensions rather than body mass alone. A study by Nevill et al. demonstrated that handgrip strength in youth is most appropriately normalized through a combination of height and mass using allometric models, and that traditional ratio methods may misrepresent size-independent strength differences. In this context, while ratio scaling remains practical and interpretable for large field studies, it should be viewed as a limitation due to its potential to oversimplify the body size–strength relationship^21^.

### Muscular strength: absolute versus relative measures

In the present study, obese children demonstrated higher absolute handgrip strength compared with non-obese children. Although obese children demonstrated higher absolute handgrip strength compared with non-obese peers, this should be interpreted with caution given that body composition was not directly measured. Without objective assessment tools such as DXA or bioelectrical impedance, we cannot infer increased lean mass. It is plausible that greater absolute strength in obese children may reflect adaptations to carrying a higher habitual load during daily activities. Chronic exposure to increased body mass may act as a repetitive mechanical stimulus, leading to neuromuscular adaptations that enhance force production capacity in specific muscle groups involved in weight-bearing tasks. Recent research suggests that obese youth exhibit neuromuscular adaptations related to force production, possibly attributable to the mechanical demands of sustained excess mass as a form of habitual loading, rather than increased lean mass per se. These adaptations may involve changes in central neural drive and voluntary activation that contribute to greater force output in weight-bearing muscles of obese adolescents^22,23^. Nonetheless, in the absence of direct body composition measures, the relative contributions of fat mass, lean mass, and frame size to absolute strength remain uncertain.

However, when muscular strength was expressed relative to body weight, obese children exhibited significantly lower relative strength. This distinction is critical, as relative muscular strength more accurately reflects functional capacity and the ability to perform daily and recreational activities^14^. Reduced relative strength may limit participation in physical activity, contribute to early fatigue, and increase the risk of musculoskeletal discomfort or injury, thereby reinforcing sedentary behavior and perpetuating obesity^15^. Recent systematic reviews emphasize that relative muscular fitness, rather than absolute strength, is more strongly associated with favorable cardiometabolic profiles in youth^19^. Therefore, reliance on absolute strength measures alone may mask functional impairments in obese children and underestimate their fitness-related health risks.

Although obese children demonstrated statistically higher absolute handgrip strength, the magnitude of the between-group difference (~ 2.3–2.4 kg) corresponded to a small-to-moderate effect size (Cohen’s d ≈ 0.4). In clinical terms, such a difference may have limited standalone functional relevance, particularly given the natural variability in adolescent strength development related to growth and maturation. In contrast, relative muscular strength exhibited a large effect size and demonstrated stronger associations with aerobic capacity, suggesting that strength relative to body mass is likely more functionally meaningful than absolute strength alone. Therefore, while absolute handgrip differences reached statistical significance, their clinical importance should be interpreted cautiously, with greater emphasis placed on relative strength measures when evaluating pediatric functional fitness.

### Association between aerobic capacity and muscular strength

The findings indicate that both adiposity and functional muscular performance are independently associated with aerobic fitness; however, their shared dependence on body mass necessitated separate analytical modeling. The stronger predictive value observed for relative muscular strength compared with absolute strength suggests that muscle quality and functional strength relative to body mass may be more important determinants of aerobic performance than absolute force production alone. This reinforces the importance of targeting improvements in relative strength rather than focusing solely on increasing absolute muscle output in pediatric fitness interventions. A key finding of this study was the significant positive association between aerobic capacity and muscular strength in both obese and non-obese children. Notably, stronger correlations were observed between aerobic capacity and relative muscular strength compared with absolute strength. These findings are consistent with previous studies reporting that children with higher muscular fitness tend to exhibit better cardiorespiratory performance^24,25^.

Several physiological mechanisms may explain this relationship. Skeletal muscle plays a central role in oxygen uptake and utilization during aerobic exercise, and greater muscular strength may reflect better muscle quality, mitochondrial function, and oxidative capacity^26^. Additionally, higher relative strength may improve movement economy, allowing children to perform aerobic tasks with lower relative effort, thereby enhancing endurance performance. The stronger associations observed in non-obese children may reflect fewer biomechanical and metabolic constraints compared with obese children. Nonetheless, the presence of significant associations in both groups suggests that improving muscular strength — particularly relative strength — may positively influence aerobic capacity irrespective of weight status. This finding supports the inclusion of resistance-based activities in physical activity interventions targeting obese youth^27^.

A methodological consideration of this study relates to the use of handgrip strength as a proxy for overall muscular fitness when examining its association with shuttle run performance. Handgrip strength primarily reflects upper-body isometric force, whereas the 20-m shuttle run predominantly assesses lower-body dynamic aerobic capacity. However, handgrip strength is widely recognized as a valid global indicator of general muscular fitness and neuromuscular function in pediatric populations^11,14^. Muscular fitness in youth is influenced by systemic physiological factors — including muscle quality, motor unit recruitment efficiency, and metabolic characteristics — which are not strictly muscle-group specific. These underlying attributes may partly explain the observed association with aerobic capacity, as skeletal muscle oxidative capacity, mitochondrial function, and movement economy contribute to both strength expression and endurance performance^25,26^. Previous research has consistently demonstrated positive relationships between general muscular fitness and aerobic capacity in children and adolescents, suggesting shared physiological and developmental determinants rather than direct mechanical coupling between specific muscle groups^11^. Importantly, maturation and adiposity remain plausible confounders, as both muscular strength and aerobic capacity are strongly influenced by growth-related changes in body composition and hormonal status^12^. Therefore, the present findings should be interpreted as reflecting associations between global fitness domains rather than implying that upper-body isometric strength directly predicts lower-body aerobic performance.

It is also important to consider that pubertal maturation may partially mediate or confound the observed relationships. Maturation-related increases in lean mass and neuromuscular coordination could strengthen associations between handgrip strength and aerobic capacity independent of adiposity. Without direct assessment of biological maturation, the extent to which developmental stage influenced these findings cannot be determined. Importantly, the cross-sectional nature of this study precludes inference of causality or temporal directionality between muscular strength and aerobic capacity. Although significant associations were observed, it cannot be determined whether greater relative handgrip strength contributes to higher aerobic capacity, whether higher aerobic fitness promotes muscular development, or whether both are influenced by shared underlying factors such as maturation, habitual physical activity, or body composition. Longitudinal and interventional studies are required to clarify causal pathways.

### Implications for practice and public health

The findings of this study have important implications for pediatric health promotion and obesity management. Reduced aerobic capacity and impaired relative muscular strength in obese children suggest that excess body mass limits both cardiorespiratory and functional performance. Traditional interventions often emphasize aerobic exercise alone; however, emerging evidence supports the inclusion of muscular strength training as part of comprehensive fitness programs for children and adolescents^28,29^. Clinicians, physiotherapists, and physical education professionals should consider incorporating strength-based activities that improve muscular performance relative to body weight, in addition to traditional aerobic exercise, when designing pediatric fitness and obesity intervention programs. Such an approach may enhance movement efficiency, reduce exercise intolerance, and facilitate greater participation in physical activity, particularly among children with obesity. School-based and community programs in Saudi Arabia may benefit from structured, age-appropriate resistance training as part of comprehensive strategies to improve youth fitness and long-term health outcomes. In the Saudi context, where physical inactivity among children is prevalent, school-based programs incorporating both aerobic and resistance exercises may represent a feasible and culturally appropriate approach to improving physical fitness and reducing obesity-related health risks^9,30^.

### Limitations

This study has several important limitations. First, the cross-sectional design precludes determination of causality or temporal relationships between aerobic capacity and handgrip strength. Observed associations should therefore be interpreted as correlational rather than causal. Second, pubertal maturation was not directly assessed. The age range of 10–15 years encompasses substantial variability in biological maturation, which strongly influences lean mass, hormonal status, neuromuscular development, and aerobic capacity. Although chronological age was included as a covariate in regression models, age adjustment does not fully account for differences in pubertal stage. The absence of maturation assessment (e.g., Tanner staging or maturity offset) may therefore have introduced residual confounding. Third, the use of the Léger equation to estimate VO₂max from shuttle run performance. Because the equation is based on a mass-dependent performance variable, it may underestimate aerobic capacity in children with BMI ≥ 95th percentile due to the increased metabolic and mechanical cost of moving excess body mass. Direct laboratory measurement of VO₂max or alternative non–mass-dependent assessments would provide a more precise evaluation of aerobic fitness in this population. Fourth, although separate regression models were constructed to avoid mathematical coupling between BMI and relative handgrip strength, shared variance related to body mass remains a potential concern. Multicollinearity diagnostics were within acceptable limits; however, statistical independence does not eliminate conceptual overlap between weight-derived variables. Future studies using direct measures of body composition (e.g., DXA) and alternative scaling approaches may provide greater clarity. Finally, muscular strength was assessed using handgrip dynamometry, which reflects upper-limb isometric strength and may not fully represent lower-limb strength relevant to running performance. Future longitudinal and intervention studies are warranted to examine causal pathways and evaluate the effectiveness of combined aerobic and strength training interventions in Saudi children.

## Conclusion

Obese children aged 10–15 years in the Riyadh region exhibited reduced aerobic capacity and lower relative handgrip strength despite higher absolute strength. Both adiposity and relative muscular strength were independently associated with aerobic fitness. These findings highlight the importance of integrating resistance (strength) training alongside aerobic exercise in pediatric obesity programs. Improving strength relative to body mass may enhance functional capacity and physical self-confidence, potentially promoting sustained participation in physical activity. Integrated school- and community-based interventions in Saudi Arabia should therefore incorporate both aerobic and resistance exercises to optimize youth fitness and reduce obesity-related health risks.

## Data Availability

The datasets used and/or analyzed during the current study available from the corresponding author on reasonable request.
